# SERPINE1 and its co-expressed genes are associated with the progression of clear cell renal cell carcinoma

**DOI:** 10.1186/s12894-023-01217-6

**Published:** 2023-03-23

**Authors:** Lingyu Guo, Tian An, Ziyan Wan, Zhixin Huang, Tie Chong

**Affiliations:** 1 Department of Medicine, Xi’an Jiaotong University, Xi’an, China; 2 Department of Dermatology and Plastic Surgery, The Second Affiliated Hospital of Shaanxi University of Traditional Chinese Medicine, Xianyang, China; 3 Department of Urology, The Second Affiliated Hospital of Xi’an Jiaotong University, 157 West Fifth Road, Xi’an, 710000 China

**Keywords:** SERPINE1, ccRCC, Biomarker, Prognosis, Tumor microenvironment

## Abstract

**Background:**

Clear cell renal cell carcinoma(ccRCC) is a frequently occurring malignant tumor of the urinary system. Despite extensive research, the regulatory mechanisms underlying the pathogenesis and progression of ccRCC remain largely unknown.

**Methods:**

We downloaded 5 ccRCC expression profiles from the Gene Expression Omnibus (GEO) database and obtained the list of differentially expressed genes (DEGs). Using String and Cytoscape tools, we determined the hub genes of ccRCC, and then analyzed their relationship with ccRCC patient survival. Ultimately, we identified SERPINE1 as a prognostic factor in ccRCC. Meanwhile, we confirmed the role of SERPINE1 in 786-O cells by cell transfection and in vitro experiments.

**Results:**

Our analysis yielded a total of 258 differentially expressed genes, comprising 105 down-regulated genes and 153 up-regulated genes. Survival analysis of SERPINE1 expression in The Cancer Genome Atlas (TCGA) confirmed its association with the increase of tumor grade, lymph node metastasis, and tumor stage, as well as with shorter survival. Furthermore, we found that SERPINE1 expression levels were associated with CD8 + T cells, CD4 + T cells, B cells, macrophages, neutrophils, and dendritic cells. Cell experiments showed that knockdown SERPINE1 expression could inhibit the proliferation, migration and invasion of ccRCC cells. Among the co-expressed genes with the highest correlation, ITGA5, SLC2A3, SLC2A14, SHC1, CEBPB, and ADA were overexpressed and associated with shorter overall survival (OS) in ccRCC.

**Conclusions:**

In this study, we identified hub genes that are strongly related to ccRCC, and highlights the potential utility of overexpressed SERPINE1 and its co-expressed genes could be used as prognostic and diagnostic biomarkers in ccRCC.

**Supplementary Information:**

The online version contains supplementary material available at 10.1186/s12894-023-01217-6.

## Introduction

Renal cell carcinoma (RCC) is a malignancy that originates in the renal epithelium, accounting for 2–3% of adult malignancies [1]. Clear cell renal cell carcinoma(ccRCC) is the most common subtype, comprising over 80% of RCC cases and is associated with the worst prognosis [2]. Although surgical resection is effective for many ccRCC patients, approximately one-third of patients still experience metastasis or postoperative recurrence, leading to ineffective treatment [3, 4]. Current clinical guidelines rely on tumor size, stage, and grade, which lack molecular biomarkers. However, even patients deemed low risk by these clinical guidelines can still experience disease progression and tumor recurrence [5]. The absence of reliable clinical biomarkers makes early diagnosis and treatment of ccRCC challenging, contributing to the high mortality rate of ccRCC [6–8]. While some studies have identified specific molecules contribute to the risk stratification of ccRCC, their clinical application may be too costly and complex [9, 10]. Nevertheless, immune-related genes have been found to be associated with ccRCC prognosis, offering a potential complement to the existing staging system [11, 12]. Thus, further revealing the biomolecular mechanism of ccRCC and identifying valuable diagnostic and prognostic biomarkers may improve the treatment of ccRCC patients.

Serpin Family E Member 1 (SERPINE1), also known as plasminogen activator inhibitor type 1 (PAI-1), is a member of the serine proteinase inhibitor (serpin) superfamily [13, 14]. It is a fibrinolytic inhibitor that involved in several human malignancies[15, 16] and is also an important component of innate antiviral immunity. Previous investigations have reported that SERPINE1 is involved in the regulatory process of various types of tumors, including gastric adenocarcinoma [14], glioma [17], bladder cancer [18], and lung cancer [19]. In gastric cancer, SERPINE1 has been found to regulate the EMT process and promote tumor progression [14], while in colon cancer, it can regulate tumor microenvironment and immune cell infiltration [20]. Furthermore, high expression of SERPINE1 in lung cancer is associated with poor prognosis [19]. However, it is still unclear whether SERPINE1 regulates the development of ccRCC. Microarray and RNA sequencing technology provide high-throughput genome sequencing data, and bioinformatics analysis enables efficient analysis of gene expression [21]. The GEO and TCGA databases provide valuable information on disease gene expression, helping to explore key signaling pathways and important molecular mechanisms of disease [22]. In this study, we selected 5 microarray data sets from the GEO database to identify DEGs and ccRCC hub genes. Using bioinformatics methods, we aimed to explore the relationship between SERPINE1 and clinical characteristics of ccRCC patients. Additionally, we performed a series of in vitro experiments to verify the biological functions of SERPINE1.

## Methods

### Screening the differentially expressed genes

We selected a total of 5 GEO databases for further research, including GSE15641, GSE16441, GSE40435, GSE53000, and GSE71963 [23–27]. To explore the molecular variation in ccRCC occurrence and development, all selected datasets included ccRCC tumor tissue samples and corresponding non-tumor tissue samples. GSE15641 from the GPL96 Affymetrix Human Genome U133A Array contains 23 normal and 32 ccRCC samples. GSE16441 from the GPL6480 Agilent-014850 Whole Human Genome Microarray contains 17 ccRCC tumors and 17 corresponding non-tumor samples. GSE40435 from GPL10558 Illumina HumanHT-12 V4.0 expression beadchip includes 101 pairs of ccRCC tumors and adjacent non-tumor renal tissue. GSE53000 from GPL6244 Affymetrix Human Gene 1.0 ST Array includes 56 ccRCC tumor samples and 6 normal samples. GSE71963 from GPL6480 Agilent-014850 Whole Human Genome Microarray includes 16 normal kidney tissues and 32 ccRCC tissues. We used the online analysis tool GEO2R (www.ncbi.nlm.nih.gov/geo/geo2r) to identify differentially expressed genes in ccRCC. Thresholds for analysis were set as *p*-value < 0.05 and |log fold change (FC)|≥ 1. Then we obtained a summary of differential genes in different datasets by drawing a Venn diagram. Finally, we identified 258 DEGs in ccRCC, of which 153 genes were up-regulated and 105 were down-regulated.

### Hub genes analysis

To evaluate the biological functions and signaling pathways of the obtained DEGs, we used the Database for Annotation, Visualization and Integrated Discovery (DAVID) (https://david.ncifcrf.gov/). The Gene Ontology (GO) and Kyoto Encyclopedia of Genes and Genomes (KEGG) enrichment analyses were performed [28–30]. To examine the correlations between hub gene proteins, the STRING database (https://cn.string-db.org/) was used to build a protein–protein interaction (PPI) network [31]. Cytoscape software (version 3.9.0) was used to visualize and analyze the PPI network [32]. The Cytoscape MCODE plug-in was used to identify key modules from the entire PPI network [33]. Subsequently, the cytoHubba plug-in was used to screen for possible hub genes from the identified genes.

To investigate the potential role of hub genes in the disease progression of ccRCC, gene expression and prognostic correlation analysis were performed. We used the Kaplan–Meier Plotter online analysis tool (https://kmplot.com/analysis/) to determine the prognostic association of hub genes with ccRCC [34].

### Immune infiltration analysis

The TIMER database (https://cistrome.shinyapps.io/timer/) was used to explore the association between SERPINE1 expression and immune infiltrates in ccRCC [35]. We also employed the cBioPortal (http://www.cbioportal.org/) and GEPIA (http://gepia.cancer-pku.cn/) web tool to calculate the relationships between SERPINE1 and tumor immune infiltration gene markers [36].

### Cell line generation and cell culture.

The 786-O cell line was obtained from the American Type Culture Collection (ATCC) and cultured in RPMI-1640 medium supplemented with 1% penicillin/streptomycin and 10% fetal bovine serum. Cells were incubated at 37 °C in a 5% CO2 incubator.

### Cell transfection

To down-regulate the expression of SERPINE1, we obtained the si-SERPINE1 sequence from GenePharma Co. Ltd, which was as follows: 5′-UGAACUUGUUGGUCUGAGCTT-3′. The negative control siRNA sequence (si-NC) was 5′-ACGUGACACGUUCGGAGAATT-3′. The transfection experiment was performed using Lipofectamine 2000 (Thermo Fisher Scientific, Inc.) following the product instructions meticulously.

### Proliferation assay

To investigate the impact of SERPINE1 on tumor cell proliferation, we conducted a CCK8 assay. 48 h post-transfection with siRNA, 786-O cells were harvested, suspended and then seeded on 96-well plates (Corning Inc, Corning, NY, USA) at a density of 1000 cells/well. At the time of the assay, CCK-8 reagent was added to each well according to the instruction. Cell viability was measured every 24 h by detecting the optical density (OD) values at the wavelength of 450 nm.

### Cell migration and invasion assay

For the cell migration and invasion assays, we used transwell chambers from Corning Inc. The transfected cells were suspended in serum-free medium and seeded into the upper chamber at a density of 2 × 10^4^ cells per well, while 600 ul of complete medium was added to the lower compartment. After incubating the cells for 24 h in a cell incubator, we used 4% paraformaldehyde to fix the invaded cells for 30 min, followed by staining with 0.1% crystal violet for 20 min. Residual cells that did not migrate were carefully removed. We calculated and photographed the cells under a microscope. For the cell invasion experiment, the bottom of the transwell chamber was covered with matrigel glue before cell seeding, and the remaining steps were the same as those for the cell migration experiment.

### Wound-healing assay

After transfection for 48 h, the 786-O cells were harvested and seeded into a 6-well plate at a density of 5 × 10^4^ cells/ml. When the cells reached more than 80% confluence, a straight wound was created in the center of each well using a 200 μl pipette tip. The cells were then washed twice with PBS to remove any floating cells. After washing, the cells were recorded as 0 h and incubated in a medium containing 2% FBS for further culture. After 24 h, the cells were observed and photographed under a microscope to evaluate the degree of wound healing.

### SERPINE1 related genes analysis

The cBioPortal website was adopted to select SERPINE1′s co-expressed genes based on 538 samples from TCGA-KIRC [37]. UALCAN (http://ualcan.path.uab.edu/) and Kaplan–Meier plotter databases were used to further analyze the expression and prognostic value of SERPINE1 related genes in ccRCC [38].

### Statistical analysis

The statistical analyses for this study were conducted using SPSS 22.0 (SPSS, Inc.), R (version 4.0.2), and GraphPad Prism (version 8.0) software. For survival analysis, patients were divided based on the median value of gene expression. ROC curves were generated using the pROC package to evaluate the diagnostic value of these genes. The unpaired Student's t-test was employed to compare the results of the two groups. Chi-square test was used to compare clinical information between groups. Spearman's correlation analysis was used to detect correlations between genes. All experiments were performed in triplicate and presented as mean ± standard deviation (SD). A *p* < 0.05 was considered statistically significant.

## Results

### Identification of DEGs in ccRCC

**Fig. 1:**
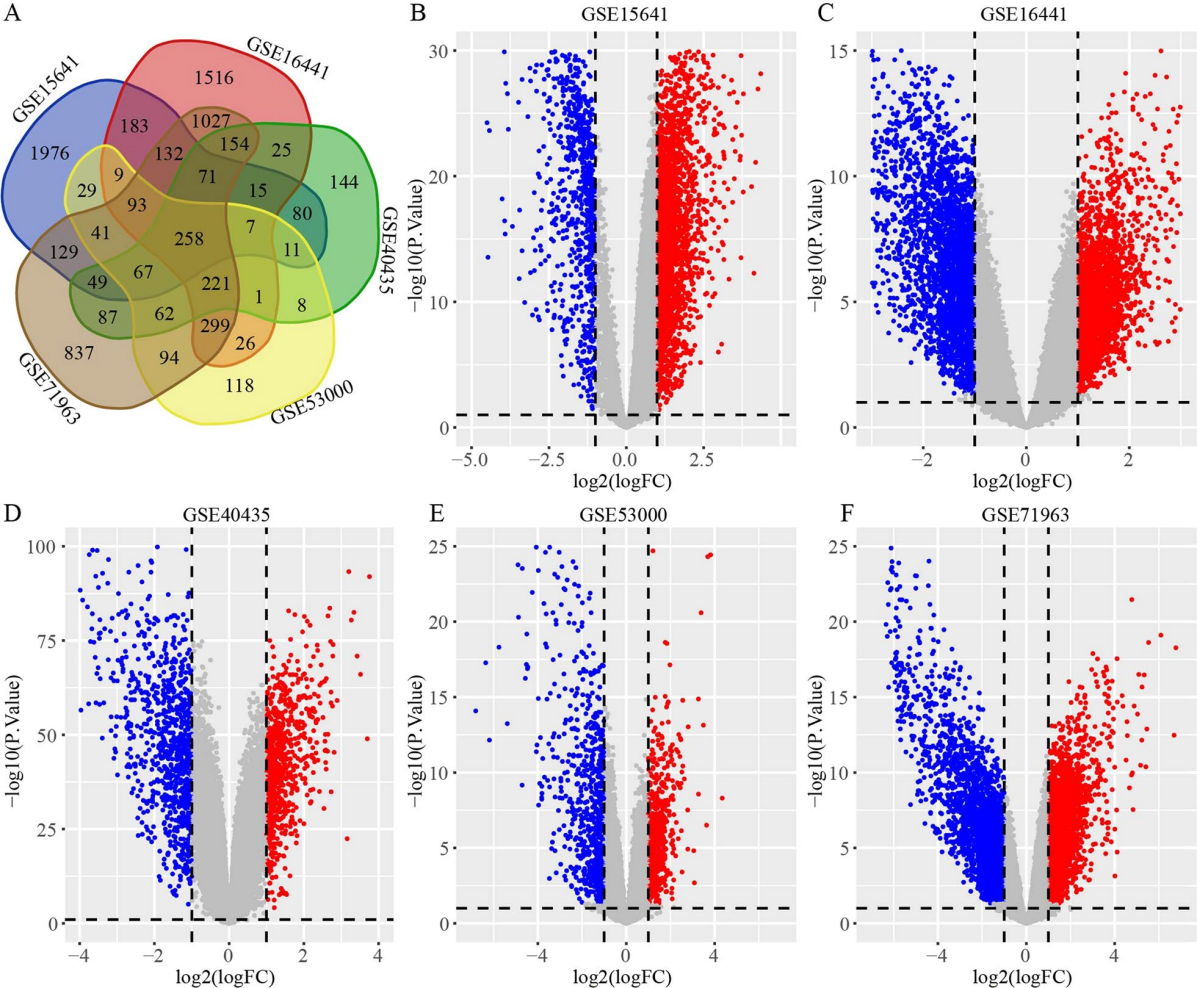
Differentially expressed genes in ccRCC. **A** Venn diagram of differentially expressed genes in different datasets. **B** GSE15641 data, **C** GSE16441 data, **D** GSE40435 data, **E** GSE53000 data, and **F** GSE71963 data. The red and blue dots represent up-regulated and down-regulated genes. The threshold for |logFC|≥ 1 and *p* < 0.05. Black points represent genes with no significant difference. FC: fold change; GEO: Gene Expression Omnibus; DEGs; differentially expressed genes

**Fig. 2:**
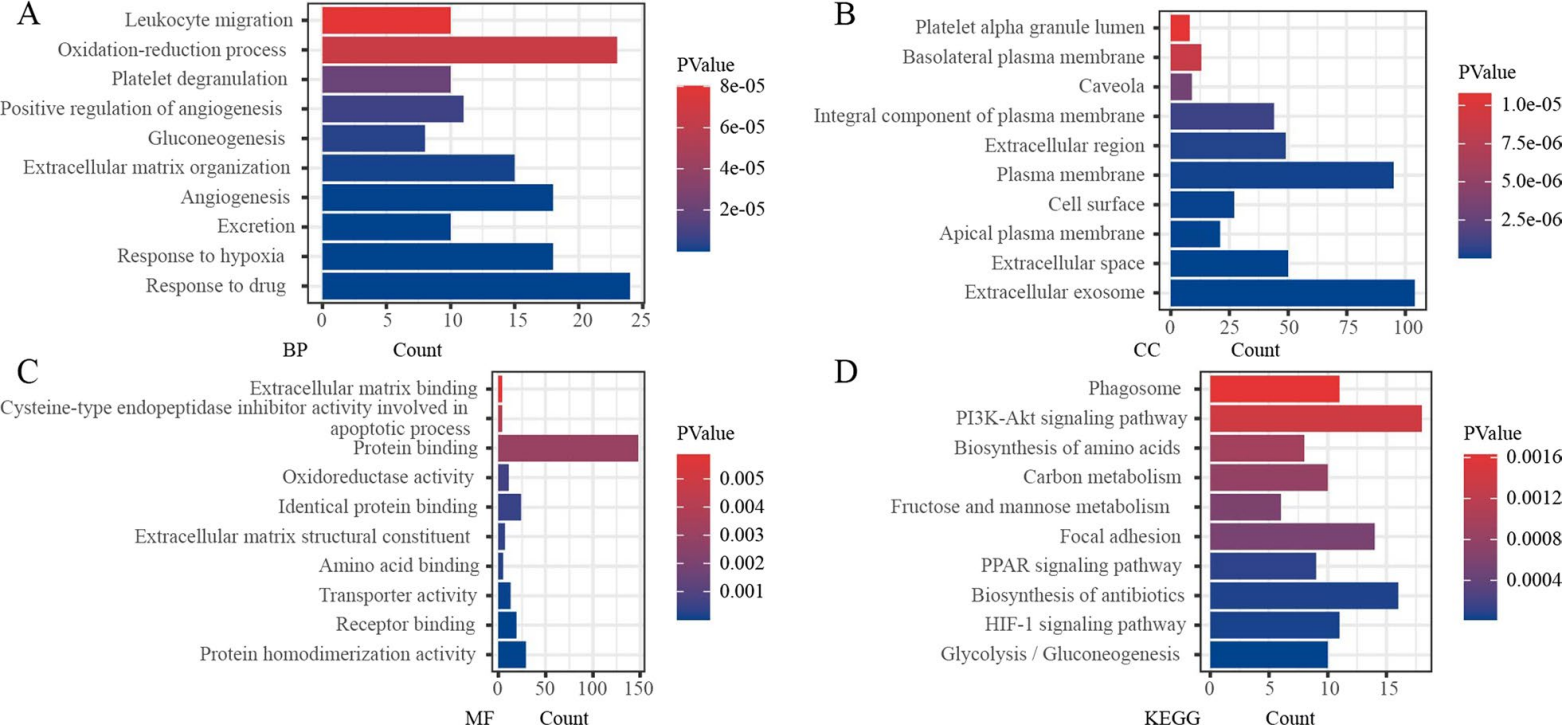
DEGs functional enrichment analysis. **A** Biological processes, **B** Cellular components, and **C** Molecular functions. **D** Kyoto Encyclopedia of Genes and Genomes (KEGG) analysis

We analyzed 5 ccRCC GEO profile datasets and identified a total of 258 DEGs, with 153 genes highly expressed in tumor tissues and 105 genes with low expression (Fig. 1A). The volcano maps illustrate the differential expression of genes in each dataset (Fig. 1B–F). To further understand the biological processes associated with these DEGs, the DAVID database was used to annotate these robust DEGs, and performed GO and KEGG analyses. In the BP portion of GO analysis, the DEGs were mainly enriched in the ‘Oxidation–reduction process’ and ‘Response to drug’ (Fig. 2A). In the CC portion of the analysis, the DEGs were primarily concentrated in the ‘Plasma membrane’ and ‘Plasma membrane’(Fig. 2B). The main molecular function performed by DEGs was’Protein binding’(Fig. 2C). Through KEGG analysis, we learned that the DEGs were mainly involved in ‘PI3K-Akt signaling pathway’, ‘Focal adhesion’, and ‘Biosynthesis of antibiotics’ (Fig. 2D). These results suggest that these DEGs play a crucial role in the disease progression of ccRCC and are closely related to the onset and recurrence of the tumor.

### Hub genes selection and analysis

**Fig. 3:**
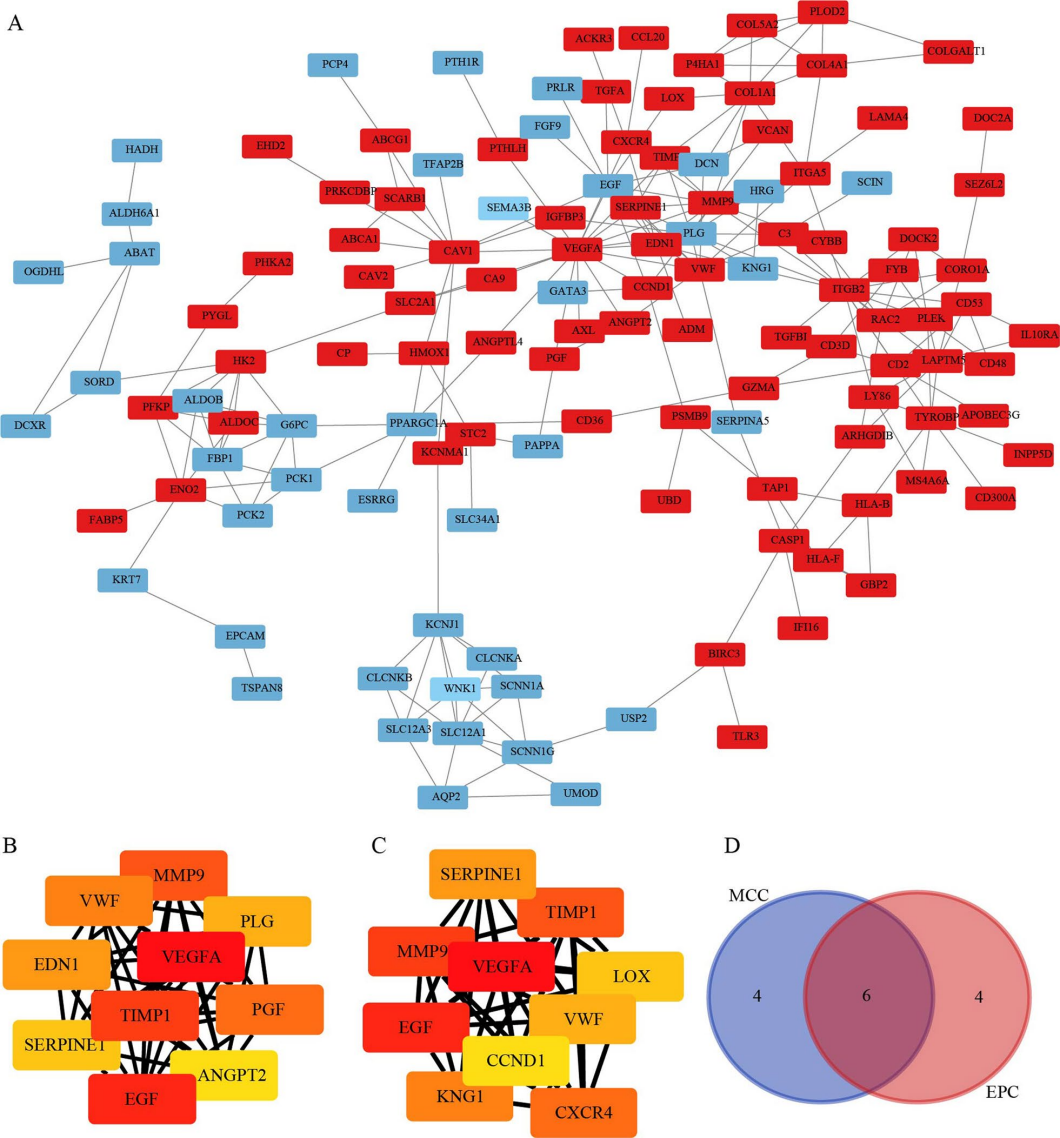
PPI network construction and hub genes analysis. **A** PPI network. **B** and **C** The top 10 hub genes were screened using the Cytoscape software plugin cytoHubba. **D** Venn diagram of hub genes. The red and blue represent up-regulated and down-regulated genes

**Fig. 4:**
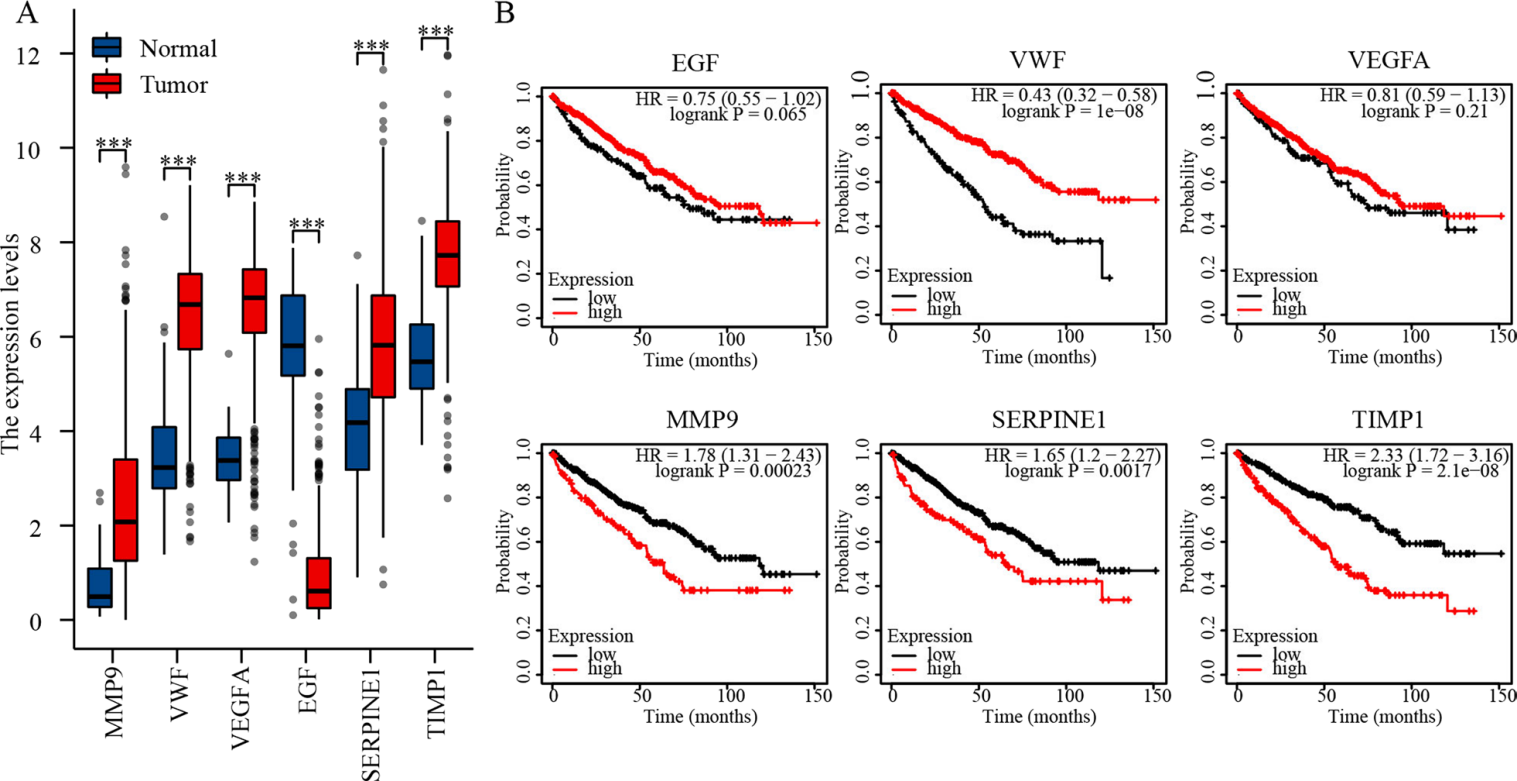
Expression and prognosis analysis of hub genes in ccRCC. **A** Expression of hub genes. **B** Relationship between hub genes expression levels and ccRCC OS. ***p < 0.001. OS, overall survival

The Cytoscape software was used to construct a PPI network of DEGs (Fig. 3A). Subsequently, CytoHubba plug-in of Cytoscape software was used to identify hub genes of ccRCC according to the MCC and EPC algorithms (Fig. 3B, C). The overlapped hub genes in the results of the two methods included VEGFA, EGF, TIMP1, MMP9, VWF, and SERPINE1(Fig. 3D). Then, we utilized the MCODE plug-in to identify the most significant module from the entire PPI network. The resulting significant module consisted of 25 nodes and 137 edges (Table 1). The expression levels and prognostic value of these 6 hub genes was studied. The expression levels of MMP9, VWF, VEGFA, SERPINE1, and TIMP1 in ccRCC tissues were higher than those in normal tissues, while the expression of EGF in tumor tissues was lower (Fig. 4A). Furthermore, higher expression of MMP9, SERPINE1, and TIMP1 suggested shorter OS in ccRCC patients, while higher expression of VWF suggested longer OS in ccRCC patients (*p* < 0.05) (Fig. 4B).

**Table 1:** Significant modules from the whole PPI network

Module	Score	Nodes	Edges	Node IDs	
1	11.417	25	137		PGF, LAPTM5, TYROBP, RAC2, CD53, VWF, PLG, FGFR1, CYBB, ARHGDIB, JAK2, EGF, HRG, SERPINE1, HMOX1, EDN1, CORO1A, PLEK, CXCR4, TIMP1, ANGPT2, FGF1, ITGB2, CD48, IL10RA
2	6.462	14	42		ANGPTL4, PPARGC1A, ENO2, HK2, FBP1, PFKP, GK, PCK2, PYGL, ALDOC, HMGCS2, SCD, PCK1, HADH
3	6	11	30		CP, LOX, STC2, LGALS1, CCND1, CDK3, MMP9, DCN, ESR1, CAV1, CA9
4	5.778	10	26		SLC12A3, CLCNKA, NPHS2, UMOD, SCNN1G, KCNJ1, SCNN1A, SLC34A1, CLCNKB, WNK1
5	5	13	30		VCAN, TGFBI, C3, CCL20, P4HA1, COL1A1, COL4A1, IGFBP3, PTGER3, ITGA5, COL5A2, ACKR3, PLOD2
6	4.8	6	12		GBP2, TAP1, PSMB9, HLA-F, FCGR1B, HLA-B
7	3.714	8	13		PCLAF, TYMS, TOP2A, SLC1A3, NUSAP1, PVALB, CALB1, FABP7
8	3	3	3		CAVIN3, EHD2, CAV2
9	3	3	3		SLCO2B1, ABCC3, SLC22A6
10	3	3	3		GABARAPL1, ANK3, ANK2

Metabolic reprogramming is a complex process that involves various metabolic pathways such as aerobic glycolysis, fatty acid metabolism, and the utilization of tryptophan, glutamine, and arginine [39]. Enrichment analysis of the DEGs also suggested that tumor metabolic reprogramming plays an important role in ccRCC. Among the above three genes, we focused on SERPINE1 due to its reported association with tumor metabolism [40]. However, the specific mechanism of how SERPINE1 regulating the progression of ccRCC is still not well studied.

Correlation between SERPINE1 expression and ccRCC progression.

**Fig. 5:**
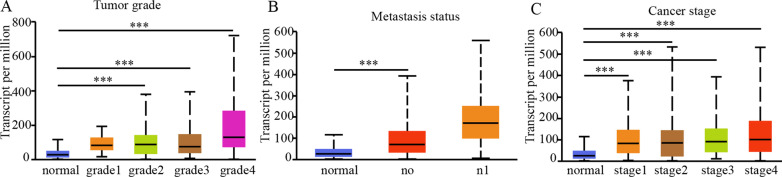
Correlation between SERPINE1 expression level and clinicopathological parameters. Correlation between SERPINE1 expression level and **A** tumor grade, **B** metastasis status, **C** cancer stage in ccRCC samples (ULCAN). ***p < 0.001.

To further explore the role of SERPINE1 in ccRCC progression, the relationship between SERPINE1 expression and other clinicopathological parameters of ccRCC were studied. The expression of SERPINE1 increased gradually with the increase of tumor grade, lymph node metastasis, and tumor stage (Fig. 5A–C). Further analysis of TCGA-KIRC data revealed that the expression level of SERPINE1 was significantly associated with patients' gender, T stage, N stage, pathologic stage, and histological grade (Table 2).

**Table 2:** Clinical information of patients with clear cell renal cell carcinoma in the TCGA

Characteristics	Low expression of SERPINE1	High expression of SERPINE1	*p-*value
n	270	271	
Age, n (%)			0.111605111
< = 60	125 (23.1%)	144 (26.6%)	
> 60	145 (26.8%)	127 (23.5%)	
Gender, n (%)			8.16029E-06
Female	118 (21.8%)	69 (12.8%)	
Male	152 (28.1%)	202 (37.3%)	
Pathologic T stage, n (%)			0.009964032
T1&T2	189 (34.9%)	161 (29.8%)	
T3&T4	81 (15%)	110 (20.3%)	
Pathologic N stage, n (%)			0.018326564
N0	134 (51.9%)	108 (41.9%)	
N1	4 (1.6%)	12 (4.7%)	
Pathologic M stage, n (%)			0.24744979
M0	215 (42.3%)	214 (42.1%)	
M1	34 (6.7%)	45 (8.9%)	
Pathologic stage, n (%)			0.015713843
Stage I&Stage II	179 (33.3%)	153 (28.4%)	
Stage III&Stage IV	89 (16.5%)	117 (21.7%)	
Histologic grade, n (%)			0.008439461
G1&G2	139 (26.1%)	111 (20.8%)	
G3&G4	125 (23.5%)	158 (29.6%)	

### Relationship between SERPINE1 and immune characteristics

**Fig. 6:**
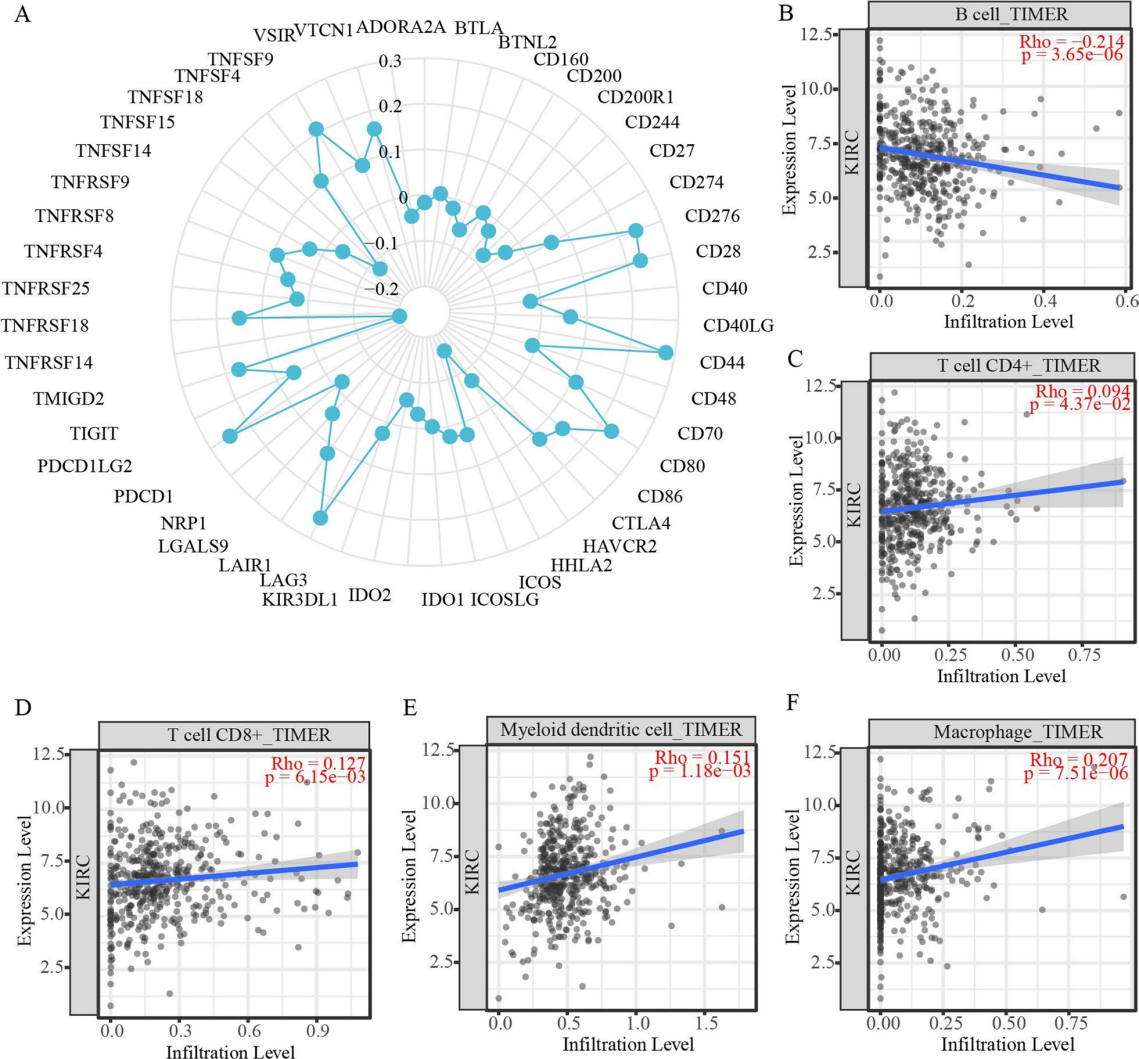
Correlation analysis of SERPINE1 expression with immune checkpoint genes and tumor immune-associated cells. **A** Correlation analysis between SERPINE1 expression levels and immune checkpoint gene levels in ccRCC. **B**–**F** SERPINE1 expression was positively closely related with B cells, CD4 + T cells, CD8 + T cells, dendritic cells, and macrophages in ccRCC (TIMER)

In recent years, immune checkpoint inhibitors have been considered effective against a wide range of tumors, and research on tumor immune checkpoints has provided many important targets for tumor immunotherapy [41]. To explore the significance of SERPINE1 in tumor immunotherapy, we investigated the relationship between SERPINE1 expression and more than 40 key immune-related genes. Interestingly, in ccRCC, SERPINE1 expression levels were significantly correlated with 20 of our selected immune checkpoint marker genes, including CD86, TNFRSF14, TNFRSF18, and CD80 (Fig. 6A). Therefore, these results show that the SERPINE1 gene may play an important role in tumor immunity. According to the TIMER database, we found that SERPINE1 in ccRCC affected tumor-infiltrating immune cells. We first analyzed the correlation between SERPINE1 expression and tumor-infiltrating immune cells, which suggested that the SERPINE1 was significantly correlated with the immune infiltration of ccRCC (Fig. 6B–F). As shown in Fig. 6B, the expression of SERPINE1 was negatively correlated with B cells (R =  − 0.214, *p* = 3.65e − 06). In addition, the expression of SERPINE1 was significantly positively correlated with various immune cell infiltrates, including CD4 + T cells (R = 0.094, *p* = 4.37e − 02), CD8 + T cells (R = 0.127, *p* = 6.15e − 03), myeloid dendritic cells (R = 0.151, *p* = 1.18e − 03), and macrophages (R = 0.207, *p* = 7.51e − 6). The table shows the correlation between SERPINE1 and immune cell-related markers on the cBioPortal website (Table 3). Therefore, SERPINE1 may play an important role in regulating the immune cell infiltration in ccRCC.

**Table 3:** Correlation analysis between SERPINE1 and biomarkers of immune cells in ccRCC determined by GEPIA database

Immune cell	Biomarker	Spearman's Correlation	*p*-value
B cell	CD19	0.259592948	1.13E-09
	CD79A	0.224938142	1.49E-07
CD8 + T cell	CD8A	0.110395509	0.010683103
	CD8B	0.07150304	0.098827444
CD4 + T cell	CD4	0.247484445	6.79E-09
M1 macrophage	NOS2	0.038746875	0.371526712
	IRF5	− 0.100882922	0.019714615
	PTGS2	0.335569557	1.61E-15
M2 macrophage	CD163	0.446177803	1.74E-27
	VSIG4	0.294943278	3.53E-12
	MS4A4A	0.347265227	1.40E-16
Neutrophil	CEACAM8	− 0.022888648	0.597672419
	ITGAM	0.116464119	0.007056392
	CCR7	0.309636063	2.50E-13
Dendritic cell	HLA-DPB1	0.091357962	0.034805998
	HLA-DQB1	0.053522596	0.216900999
	HLA-DRA	0.108000281	0.012518625
	HLA-DPA1	0.08194073	0.05845524
	CD1C	0.01007617	0.816301262
	NRP1	0.256830988	1.72E-09
	ITGAX	0.145059254	0.000773698
Monocyte	CSF1R	0.180369596	2.7562E-05
	CD86	0.213463917	6.40E-07
Treg	FOXP3	0.302728148	8.83E-13
	CCR8	0.222884005	1.95E-07
	TGFB1	0.478831915	5.89E-32

### Analysis of SERPINE1 related genes in ccRCC

**Fig. 7:**
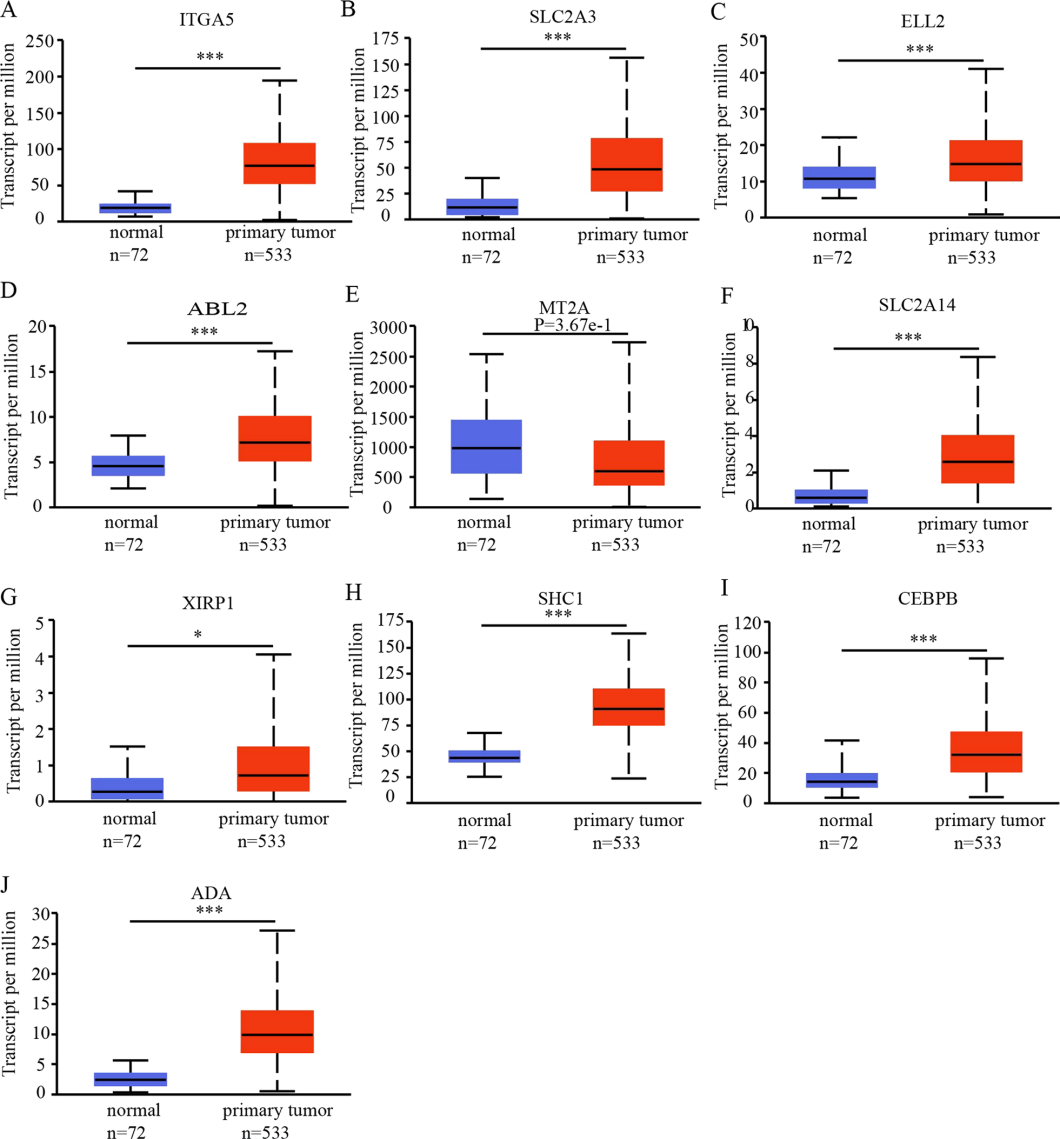
Expression of SERPINE1-related genes in ccRCC. **A**–**J** The expression levels of ITGA5, SLC3A3, ELL2, ABL2, MT2A, SLC2A14, XIRP1, SHC1, CEBPB, and ADA in ccRCC (ULCAN). ***p < 0.001

**Fig. 8:**
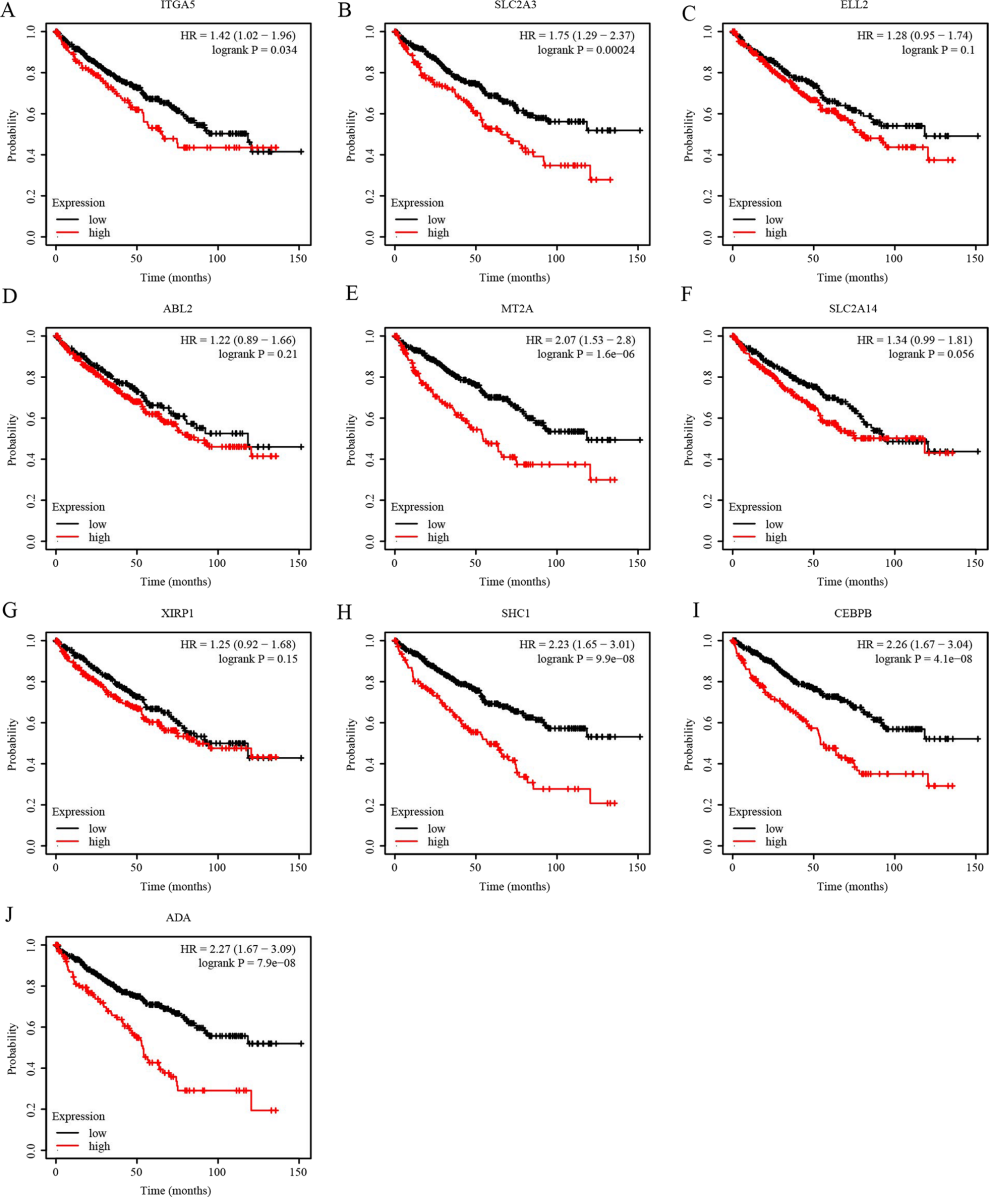
The prognostic value of SERPINE1-related genes. **A**–**J** The expression levels of ITGA5, SLC3A3, ELL2, ABL2, MT2A, SLC2A14, XIRP1, SHC1, CEBPB, and ADA were correlated with OS in ccRCC (Kaplan–Meier Plotter). OS, overall survival

We conducted an analysis of TCGA-KIRC data stored in the cBioPortal database to identify genes associated with SERPINE1 expression in ccRCC. From this analysis, we selected the 10 genes that were most highly associated with SERPINE1 expression, namely ITGA5, SLC2A3, ELL2, ABL2, MT2A, SLC2A14, XIRP1, SHC1, CEBPB, and ADA (Table 4). Of these genes, ITGA5 exhibited the highest correlation with SERPINE1 expression, with a Pearson’s Correlation *R* = 0.69 and Spearman’s Correlation *R* = 0.66. Furthermore, we analyzed the biological functions of these co-expressed genes and found that they may serve as biomarkers involved in tumor occurrence and progression. To investigate their expression and prognostic value in ccRCC and normal renal tissues, we explored the expression and survival data in the UALCAN and Kaplan–Meier plotter databases. The results showed that 9 of the 10 co-expressed genes, including ITGA5, SLC2A3, ELL2, ABL2, SLC2A14, XIRP1, SHC1, CEBPB, and ADA, were significantly upregulated in ccRCC tissues (Fig. 7A–J). In addition, the expression levels of 6 out of the 10 co-expressed genes were significantly correlated with disease OS, including ITGA5, SLC2A3, MT2A, SHC1, CEBPB, and ADA (Fig. 8A–J).

**Table 4:** Co-expression genes of SERPINE1 in ccRCC by cBioPortal

Correlated Gene	Cytoband	Pearson's Correlation	Spearman's Correlation	*p*-Value	q-Value
ITGA5	12q13.13	0.69	0.656793004	3.19E-67	6.39E-63
SLC2A3	12p13.31	0.62	0.605776476	8.61E-55	8.62E-51
ELL2	5q15	0.62	0.604081202	2.04E-54	1.36E-50
ABL2	1q25.2	0.58	0.601120857	9.10E-54	4.56E-50
MT2A	16q13	0.62	0.592322331	7.07E-52	2.83E-48
SLC2A14	12p13.31	0.53	0.591607955	1.00E-51	3.34E-48
XIRP1	3p22.2	0.52	0.580586879	1.91E-49	5.47E-46
SHC1	1q21.3	0.6	0.577511698	8.00E-49	2.00E-45
CEBPB	20q13.13	0.58	0.569524321	3.06E-47	6.82E-44
ADA	20q13.12	0.59	0.567509726	7.56E-47	1.51E-43

**Fig. 9:**
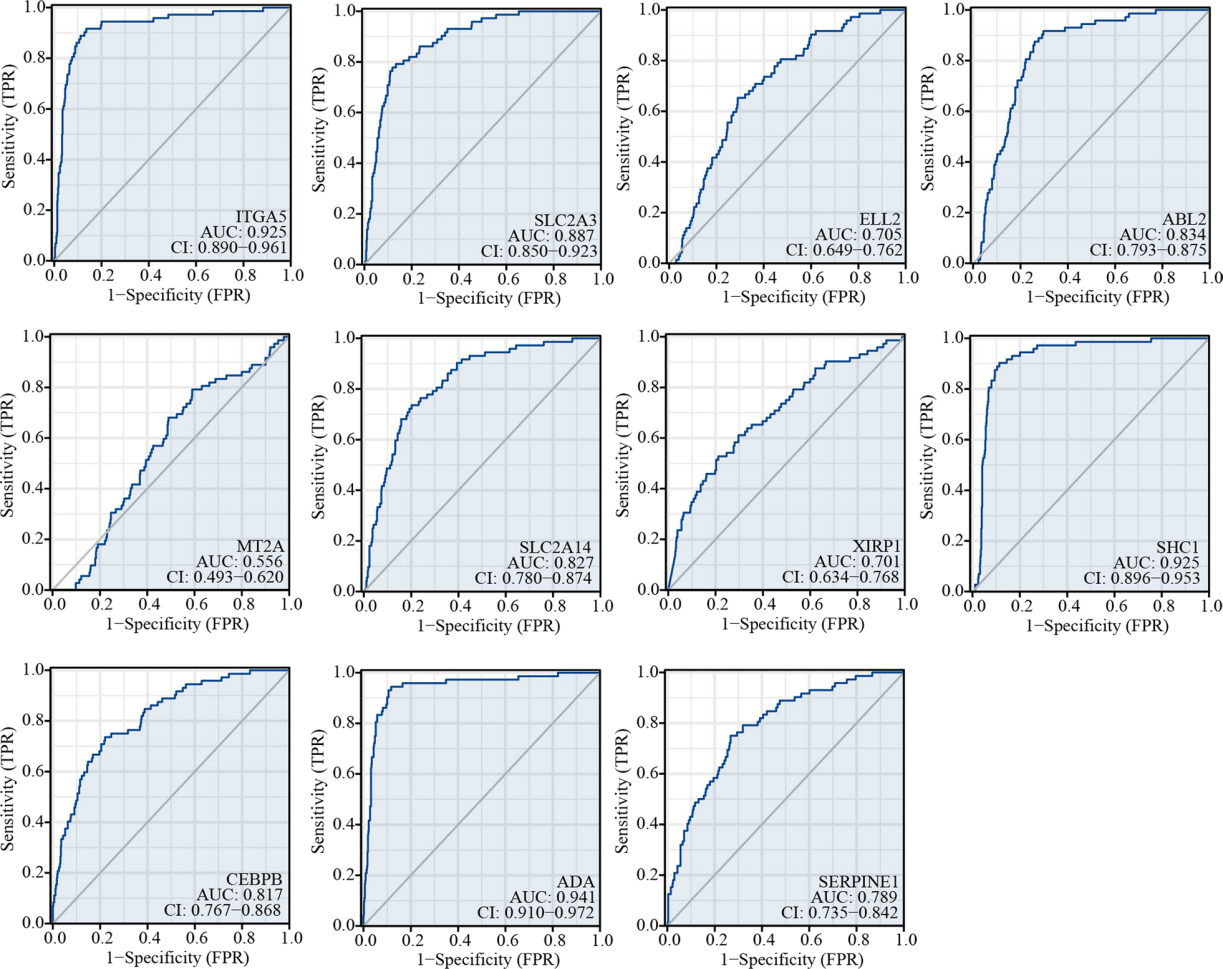
The diagnostic value of SERPINE1-related genes. ROC, receiver operating characteristic curve. AUC, area under the curve

To evaluate the diagnostic value of SERPINE1 and its co-expressed genes in ccRCC, we conducted receiver operating characteristic (ROC) curve analysis on the gene expression data downloaded from TCGA. The results demonstrated that SERPINE1 expression had high diagnostic value in ccRCC with an area under the curve (AUC) of 0.789 (Fig. 9). Moreover, the expression levels of SERPINE1 co-expressed genes also showed high diagnostic significance with ITGA5(AUC = 0.925), SLC2A3(AUC = 0.887), ELL2(AUC = 0.705), ABL2(AUC = 0.834), MT2A(AUC = 0.556), SLC2A14(AUC = 0.827), XIRP1(AUC = 0.701), SHC1(AUC = 0.925), CEBPB(AUC = 0.817), and ADA(AUC = 0.941). In summary, nearly all of the related genes (9 out of 10) exhibited promising diagnostic potential.

### Knockdown of SERPINE1 influenced ccRCC cell proliferation, migration, and invasion

**Fig. 10:**
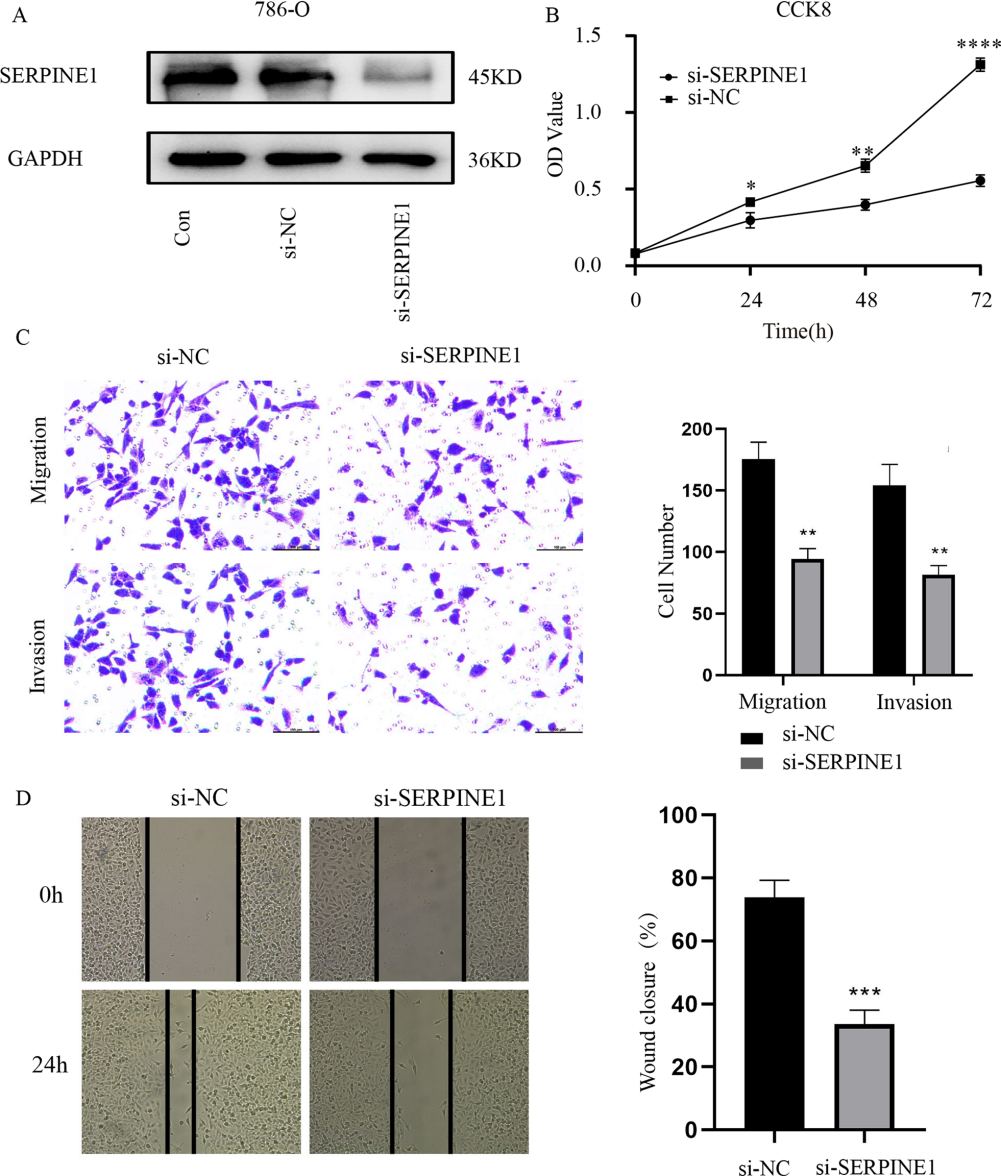
Analysis of SERPINE1 in ccRCC. **A** Western blot analysis of SERPINE1 expression in 786-O cells after transfection of SERPINE1 siRNA. **B** OD value of si-SERPINE1 cells. **C** Wound healing assay of si-SERPINE1 cells. **D** Migratory and invasive potential of si-SERPINE1 cells. *p < 0.05, **p < 0.01, ***p < 0.001

After transfecting 786-O cells with si-RNA transfection for 72 h, cell proteins were extracted for a Western Blot experiment to detect the expression level of SERPINE1 protein. The results indicated that SERPINE1 expression level in the si-RNA transfected 786-O cells was significantly lower than that in the si-NC group (Fig. 10A). In this study, CCK8 assay was used to evaluate the effect of knockdown SERPINE1 expression on the proliferation of 786-O cells. The results showed that the proliferation ability of 786-O cells decreased with the decrease of SERPINE1expression (*p* < 0.001, Fig. 10B). These results suggest that low SERPINE1 expression can inhibit the proliferation of ccRCC cells. To explore the effect of SERPINE1 expression on the migration and invasion ability of ccRCC cells, a transwell experiment was designed and implemented. The results suggested that the migration and invasion of 786-O cells were significantly reduced after SERPINE1 expression was down-regulated (Fig. 10C). In addition, cell scratch assay results showed that downregulation of SERPINE1 reduced the wound healing ability of 786-O cells (Fig. 10D). Overall, our in vitro experiments indicated that the high expression of SERPINE1 in ccRCC was associated with the development and metastasis of ccRCC.

## Discussion

In this study, we identified hub genes in ccRCC that have potential as biomarkers through data mining of the GEO database. We then analyzed the expression level and prognostic value of these hub genes in ccRCC tissues based on the TCGA database. Our results showed that three genes (MMP9, SERPINE1, and TIMP1) were overexpressed in ccRCC, and high expression of these genes suggested poor prognosis. TIMP1 has been shown to be involved in the EMT process of ccRCC and can enhance tumor cells metastasis [42]. MMP9 can regulate the immune infiltration in ccRCC and affect tumor progression [43]. Studies have shown that SERPINE1 is involved in glucose and lipid metabolism [40, 44], which may be associated with the metabolic reprogramming of ccRCC. After combining clinical data downloaded from TCGA, we found that SERPINE1 expression was closely correlated with clinical indicators such as cancer stage and survival. Importantly, our further analysis revealed that SERPINE1 expression was associated with several immune biomarkers, indicating that SERPINE1 may have therapeutic value as a target of immunotherapy for ccRCC.

SERPINE1 is known to inhibit tissue plasminogen activator (tPA) and urokinase-type plasminogen activator (PLAU) [16]. As a PLAU inhibitor, it can participate in the regulation of cell adhesion and spreading regulatory process [45]. Although the exact role of of SERPINE1 in disease and its association with the disease are not fully understood, researchers have shown that growth factors, cytokines, and hormones can regulate its expression [46]. Higgins [47] studied the expression of SERPINE1 in squamous cell carcinoma and found that TGF-β1 induces the high expression of SERPINE1 at the early stage of the tumor, with such high expression distributed on the invasive front of the tumor. Other findings have suggested interactions between SERPINE1 and the EGFR/MEK/Rho-ROCK signaling pathway [48]. Additionally, studies have shown that high SERPINE1 expression can help cells resist programmed cell death and regulate cell survival by activating Akt and ERK signaling pathways or inhibiting Fas/FasL-dependent apoptosis [49, 50]. Despite these findings, the specific mechanism by which SERPINE1 regulates tumor remains unknown. In this study, we used si-RNA transfection to knockdown the expression of SERPINE1 in the ccRCC cell line 786-O, and we found that inhibition of SERPINE1 expression reduced the proliferation, migration, and invasion of ccRCC cells. In addition, we explored genes co-expressed with SERPINE1 in ccRCC to provide a basis for explaining the regulation ability of SERPINE1 in ccRCC.

Through co-expression analysis using the cBioPortal database, we identified a set of co-expressed genes with SERPINE1 in ccRCC. These co-expressed genes were found to be involved in transmembrane transporter activity, epidermal growth factor receptor binding, and other pathways. Interestingly, 9 out of the 10 co-expressed genes, including ITGA5, SLC2A3, ELL2, ABL2, SLC2A14, XIRP1, SHC1, CEBPB, and ADA, were significantly upregulated in ccRCC tissues. Additionally, the expression levels of 6 out of the 10 co-expressed genes were significantly correlated with disease OS, including ITGA5, SLC2A3, MT2A, SHC1, CEBPB, and ADA. Previous studies have demonstrated that these co-expressed genes play important roles in the regulation of a variety of tumors. For example, ITGA5 has been shown to ptomote the occurrence and development of colorectal cancer [51], SLC2A3 can promote macrophage infiltration by glycolysis reprogramming in gastric cancer [52]. Zhao et al. [53] study showed that MT2A promotes oxaliplatin resistance in colorectal cancer cells, SHC1 is a key driver of breast cancer initiation [54], CEBPB can regulate stemness and chemo-resistance of gastric cancer [55], and ADA tends to have a positive association with breast cancer [56]. Based on these findings, we believe that SERPINE1 and its co-expressed genes are widely involved in the occurrence and development of ccRCC, and may have high diagnostic and therapeutic value. However, further in vivo and in vitro studies are necessary to support these hypotheses and develop them as potential diagnostic and therapeutic targets for ccRCC.

There are still some limitations in this study. Firstly, clinical samples of ccRCC were not available, and thus, tissue-level expression of SERPINE1 could not be evaluated. Secondly, although some findings were validated by in vitro experiments, further experiments are necessary to strengthen the results. Thirdly, the roles of SERPINE1 co-expressed genes in ccRCC have not been experimentally confirmed. This needs to be explored and demonstrated in future studies (Additional file 1).

## Conclusion

In conclusion, this study has demonstrated the high expression of SERPINE1 in ccRCC and its correlation with various clinicopathological features, indicating the potential diagnostic and therapeutic value of SERPINE1 in ccRCC.

## Supplementary Information

**Additional file 1**. **Supplement figure 1.** Raw immunoblot data for images in Fig. 10A. (**A**). Immunoblot anti-SERPINE1 of ccRCC cells. (**B**). Immunoblot anti-GAPDH of ccRCC cells.

## Data Availability

The datasets analyzed during the current study are available in the TCGA database (https://tcga-data.nci.nih.gov/tcga/), GEO database (https://www.ncbi.nlm.nih.gov/geo/), and c-BioPortal database (http://cbioportal.org).

## References

[1] Siegel RL, Miller KD, Fuchs HE, Jemal A (2021). Cancer statistics, 2021. CA Cancer J Clin.

[2] Shao Y, Wu B, Jia W, Zhang Z, Chen Q, Wang D (2020). Prognostic value of pretreatment neutrophil-to-lymphocyte ratio in renal cell carcinoma: a systematic review and meta-analysis. BMC Urol.

[3] Lalani AA, McGregor BA, Albiges L, Choueiri TK, Motzer R, Powles T, Wood C, Bex A (2019). Systemic treatment of metastatic clear cell renal cell carcinoma in 2018: current paradigms, use of immunotherapy, and future directions. Eur Urol.

[4] Siska PJ, Beckermann KE, Rathmell WK, Haake SM (2017). Strategies to overcome therapeutic resistance in renal cell carcinoma. Urol Oncol.

[5] Ljungberg B, Bensalah K, Canfield S, Dabestani S, Hofmann F, Hora M, Kuczyk MA, Lam T, Marconi L, Merseburger AS (2015). EAU guidelines on renal cell carcinoma: 2014 update. Eur Urol.

[6] Kim SH, Park WS, Chung J (2019). Tumour heterogeneity in triplet-paired metastatic tumour tissues in metastatic renal cell carcinoma: concordance analysis of target gene sequencing data. J Clin Pathol.

[7] Bedke J, Gauler T, Grunwald V, Hegele A, Herrmann E, Hinz S, Janssen J, Schmitz S, Schostak M, Tesch H (2017). Systemic therapy in metastatic renal cell carcinoma. World J Urol.

[8] Atkins MB, Tannir NM (2018). Current and emerging therapies for first-line treatment of metastatic clear cell renal cell carcinoma. Cancer Treat Rev.

[9] Rini B, Goddard A, Knezevic D, Maddala T, Zhou M, Aydin H, Campbell S, Elson P, Koscielny S, Lopatin M (2015). A 16-gene assay to predict recurrence after surgery in localised renal cell carcinoma: development and validation studies. Lancet Oncol.

[10] Yu J, Mao W, Sun S, Hu Q, Wang C, Xu Z, Liu R, Chen S, Xu B, Chen M (2021). Identification of an m6A-related lncRNA signature for predicting the prognosis in patients with kidney renal clear cell carcinoma. Front Oncol.

[11] Liao G, Wang P, Wang Y (2021). Identification of the prognosis value and potential mechanism of immune checkpoints in renal clear cell carcinoma microenvironment. Front Oncol.

[12] Hua X, Chen J, Su Y, Liang C (2020). Identification of an immune-related risk signature for predicting prognosis in clear cell renal cell carcinoma. Aging.

[13] Zhang Q, Lei L, Jing D (2020). Knockdown of SERPINE1 reverses resistance of triplenegative breast cancer to paclitaxel via suppression of VEGFA. Oncol Rep.

[14] Yang JD, Ma L, Zhu Z (2019). SERPINE1 as a cancer-promoting gene in gastric adenocarcinoma: facilitates tumour cell proliferation, migration, and invasion by regulating EMT. J Chemother.

[15] Lopez-Legarrea P, Mansego ML, Zulet MA, Martinez JA (2013). SERPINE1, PAI-1 protein coding gene, methylation levels and epigenetic relationships with adiposity changes in obese subjects with metabolic syndrome features under dietary restriction. J Clin Biochem Nutr.

[16] Freeberg MAT, Farhat YM, Easa A, Kallenbach JG, Malcolm DW, Buckley MR, Benoit DSW, Awad HA (2018). Serpine1 Knockdown enhances MMP activity after flexor tendon injury in mice: implications for adhesions therapy. Sci Rep.

[17] Wu DM, Wang S, Wen X, Han XR, Wang YJ, Fan SH, Zhang ZF, Shan Q, Lu J, Zheng YL (2018). MircoRNA-1275 promotes proliferation, invasion and migration of glioma cells via SERPINE1. J Cell Mol Med.

[18] Sun X, Cai Y, Hu XH, Mo M, Zhao C, He W, Li YL (2020). Long noncoding RNA MAFG-AS1 facilitates bladder cancer tumorigenesis via regulation of miR-143-3p/SERPINE1 axis. Transl Cancer Res.

[19] Kong HJ, Kwon EJ, Kwon OS, Lee H, Choi JY, Kim YJ, Kim W, Cha HJ (2021). Crosstalk between YAP and TGF beta regulates SERPINE1 expression in mesenchymal lung cancer cells. Int J Oncol.

[20] Wang S, Pang L, Liu Z, Meng X (2021). SERPINE1 associated with remodeling of the tumor microenvironment in colon cancer progression: a novel therapeutic target. BMC Cancer.

[21] Yang S, Gao K, Li W (2019). Identification of hub genes and pathways in glioblastoma by bioinformatics analysis. Oncol Lett.

[22] Bris C, Goudenege D, Desquiret-Dumas V, Charif M, Colin E, Bonneau D, Amati-Bonneau P, Lenaers G, Reynier P, Procaccio V (2018). Bioinformatics tools and databases to assess the pathogenicity of mitochondrial DNA variants in the field of next generation sequencing. Front Genet.

[23] Jones J, Otu H, Spentzos D, Kolia S, Inan M, Beecken WD, Fellbaum C, Gu X, Joseph M, Pantuck AJ (2005). Gene signatures of progression and metastasis in renal cell cancer. Clin Cancer Res.

[24] Liu H, Brannon AR, Reddy AR, Alexe G, Seiler MW, Arreola A, Oza JH, Yao M, Juan D, Liou LS (2010). Identifying mRNA targets of microRNA dysregulated in cancer: with application to clear cell renal cell carcinoma. BMC Syst Biol.

[25] Wozniak MB, Le Calvez-Kelm F, Abedi-Ardekani B, Byrnes G, Durand G, Carreira C, Michelon J, Janout V, Holcatova I, Foretova L (2013). Integrative genome-wide gene expression profiling of clear cell renal cell carcinoma in Czech Republic and in the United States. PLoS ONE.

[26] Gerlinger M, Horswell S, Larkin J, Rowan AJ, Salm MP, Varela I, Fisher R, McGranahan N, Matthews N, Santos CR (2014). Genomic architecture and evolution of clear cell renal cell carcinomas defined by multiregion sequencing. Nat Genet.

[27] Takahashi M, Tsukamoto Y, Kai T, Tokunaga A, Nakada C, Hijiya N, Uchida T, Daa T, Nomura T, Sato F (2016). Downregulation of WDR20 due to loss of 14q is involved in the malignant transformation of clear cell renal cell carcinoma. Cancer Sci.

[28] Huang DW, Sherman BT, Lempicki RA (2009). Systematic and integrative analysis of large gene lists using DAVID bioinformatics resources. Nat Protoc.

[29] Kanehisa M (2002). The KEGG database. Novart Fdn Symp.

[30] Ashburner M, Ball CA, Blake JA, Botstein D, Butler H, Cherry JM, Davis AP, Dolinski K, Dwight SS, Eppig JT (2000). Gene ontology: tool for the unification of biology. The gene ontology consortium. Nat Genet.

[31] Szklarczyk D, Franceschini A, Wyder S, Forslund K, Heller D, Huerta-Cepas J, Simonovic M, Roth A, Santos A, Tsafou KP et al: STRING v10: protein-protein interaction networks, integrated over the tree of life. Nucleic Acids Res 2015, 43(Database issue):D447–452. 10.1093/nar/gku1003 PMC4383874 25352553

[32] Smoot ME, Ono K, Ruscheinski J, Wang PL, Ideker T (2011). Cytoscape 2.8: new features for data integration and network visualization. Bioinformatics.

[33] Bandettini WP, Kellman P, Mancini C, Booker OJ, Vasu S, Leung SW, Wilson JR, Shanbhag SM, Chen MY, Arai AE (2012). MultiContrast Delayed Enhancement (MCODE) improves detection of subendocardial myocardial infarction by late gadolinium enhancement cardiovascular magnetic resonance: a clinical validation study. J Cardiovasc Magn Reson.

[34] Gyorffy B (2021). Survival analysis across the entire transcriptome identifies biomarkers with the highest prognostic power in breast cancer. Comput Struct Biotec.

[35] Li T, Fu J, Zeng Z, Cohen D, Li J, Chen Q, Li B, Liu XS (2020). TIMER2.0 for analysis of tumor-infiltrating immune cells. Nucleic Acids Res.

[36] Tang Z, Li C, Kang B, Gao G, Li C, Zhang Z (2017). GEPIA: a web server for cancer and normal gene expression profiling and interactive analyses. Nucleic Acids Res.

[37] Cerami E, Gao J, Dogrusoz U, Gross BE, Sumer SO, Aksoy BA, Jacobsen A, Byrne CJ, Heuer ML, Larsson E (2012). The cBio cancer genomics portal: an open platform for exploring multidimensional cancer genomics data. Cancer Discov.

[38] Chandrashekar DS, Bashel B, Balasubramanya SAH, Creighton CJ, Ponce-Rodriguez I, Chakravarthi B, Varambally S (2017). UALCAN: a portal for facilitating tumor subgroup gene expression and survival analyses. Neoplasia.

[39] Wettersten HI, Aboud OA, Lara PN, Weiss RH (2017). Metabolic reprogramming in clear cell renal cell carcinoma. Nat Rev Nephrol.

[40] Bai L, Wang W, Xiang Y, Wang S, Wan S, Zhu Y (2021). Aberrant elevation of GDF8 impairs granulosa cell glucose metabolism via upregulating SERPINE1 expression in patients with PCOS. Mol Ther Nucleic Acids.

[41] Su Y, Fu J, Du J, Wu B (2020). First-line treatments for advanced renal-cell carcinoma with immune checkpoint inhibitors: systematic review, network meta-analysis and cost-effectiveness analysis. Ther Adv Med Oncol.

[42] Shi SN, Qin X, Wang S, Wang WF, Zhu YF, Lin Y, Zhou ZL, Shi BK, Liu XG (2020). Identification of potential novel differentially-expressed genes and their role in invasion and migration in renal cell carcinoma. Aging.

[43] Xu T, Gao S, Liu J, Huang Y, Chen K, Zhang X (2021). MMP9 and IGFBP1 regulate tumor immune and drive tumor progression in clear cell renal cell carcinoma. J Cancer.

[44] Hu Q, Peng J, Chen X, Li H, Song M, Cheng B, Wu T (2019). Obesity and genes related to lipid metabolism predict poor survival in oral squamous cell carcinoma. Oral Oncol.

[45] Yang K, Zhang S, Zhang D, Tao Q, Zhang T, Liu G, Liu X, Zhao T (2019). Identification of SERPINE1, PLAU and ACTA1 as biomarkers of head and neck squamous cell carcinoma based on integrated bioinformatics analysis. Int J Clin Oncol.

[46] Ghosh AK, Murphy SB, Kishore R, Vaughan DE (2013). Global gene expression profiling in PAI-1 knockout murine heart and kidney: molecular basis of cardiac-selective fibrosis. PLoS ONE.

[47] Higgins PJ (2014). Balancing AhR-dependent pro-oxidant and Nrf2-responsive anti-oxidant pathways in age-related retinopathy: is SERPINE1 expression a therapeutic target in disease onset and progression?. J Mol Genet Med.

[48] Freytag J, Wilkins-Port CE, Higgins CE, Higgins SP, Samarakoon R, Higgins PJ (2010). PAI-1 mediates the TGF-beta1+EGF-induced "scatter" response in transformed human keratinocytes. J Invest Dermatol.

[49] Pavon MA, Arroyo-Solera I, Tellez-Gabriel M, Leon X, Viros D, Lopez M, Gallardo A, Cespedes MV, Casanova I, Lopez-Pousa A (2015). Enhanced cell migration and apoptosis resistance may underlie the association between high SERPINE1 expression and poor outcome in head and neck carcinoma patients. Oncotarget.

[50] Yao H, He G, Chen C, Yan S, Lu L, Song L, Vijayan KV, Li Q, Xiong L, Miao X (2017). PAI1: a novel PP1-interacting protein that mediates human plasma's anti-apoptotic effect in endothelial cells. J Cell Mol Med.

[51] Yu M, Chu ST, Fei BY, Fang XD, Liu Z: O-GlcNAcylation of ITGA5 facilitates the occurrence and development of colorectal cancer. Exp Cell Res 2019, 382(2–3). 10.1016/j.yexcr.2019.06.009 31202709

[52] Yao XX, He ZK, Qin CLT, Deng XQ, Bai L, Li GX, Shi JL: SLC2A3 promotes macrophage infiltration by glycolysis reprogramming in gastric cancer. Cancer Cell Int 2020, 20(1). 10.1186/s12935-020-01599-9 PMC7552479 33061855

[53] Zhao ZC, Zhang GJ, Li WD (2020). MT2A promotes oxaliplatin resistance in colorectal cancer cells. Cell Biochem Biophys.

[54] Wright KD, Miller BS, El-Meanawy S, Tsaih SW, Banerjee A, Geurts AM, Sheinin Y, Sun Y, Kalyanaraman B, Rui H (2019). The p52 isoform of SHC1 is a key driver of breast cancer initiation. Breast Cancer Res.

[55] Wu H, Liu B, Chen Z, Li G, Zhang Z (2020). MSC-induced lncRNA HCP5 drove fatty acid oxidation through miR-3619-5p/AMPK/PGC1alpha/CEBPB axis to promote stemness and chemo-resistance of gastric cancer. Cell Death Dis.

[56] Afshar AS, Nematpour FS, Meshkani M, Khafi A (2017). Growth inhibition of human breast cancer cells and down-regulation of ODC1 and ADA genes by Nepeta binaloudensis. Rev Bras Farmacogn.

