# Supplementary material for: SERPINE1 and its co-expressed genes are associated with the progression of clear cell renal cell carcinoma
Source: BMC Urol. 2023 Mar 23;23:43. doi: 10.1186/s12894-023-01217-6 (PMC10037920; doi:10.1186/s12894-023-01217-6)
Supplement: Supplementary file 1 — Additional file 1. Supplement figure 1. Raw immunoblot data for images in Fig. 10A. (A). Immunoblot anti-SERPINE1 of ccRCC cells. (B). Immunoblot anti-GAPDH of ccRCC cells. [file 12894_2023_1217_MOESM1_ESM.pdf]

A.SERPINE1

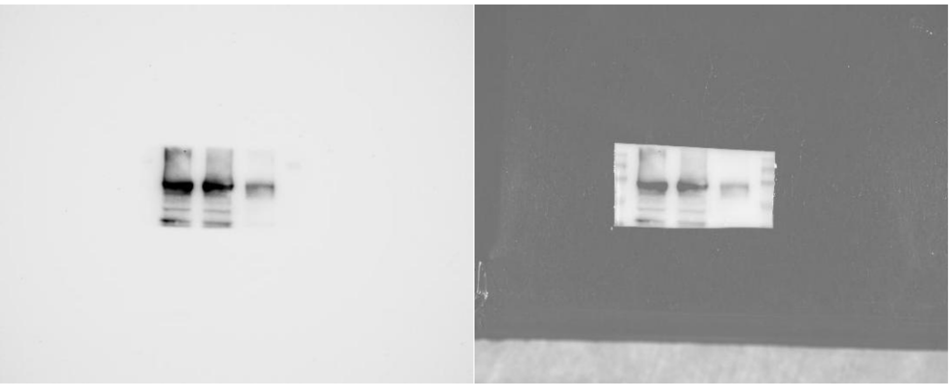

B.GAPDH

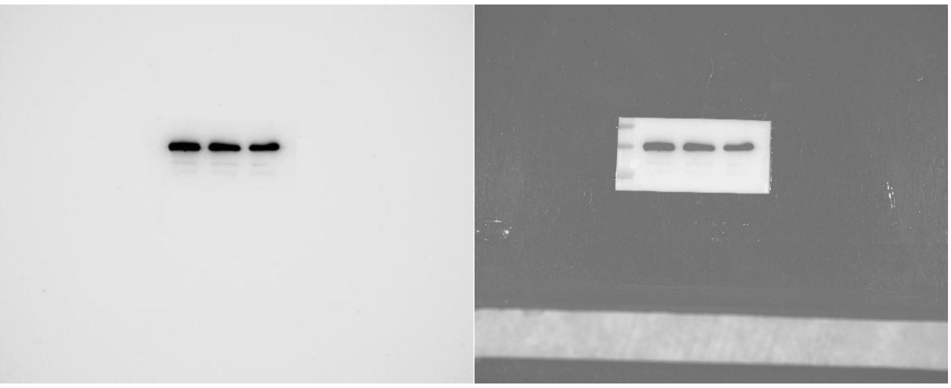

Supplement figure 1. Raw immunoblot data for images in Fig. 10A. (A).Immunoblot anti-SERPINE1 of ccRCC cells. (B).Immunoblot anti-GAPDH of ccRCC cells.
